# Advances in Low-Lactose/Lactose-Free Dairy Products and Their Production

**DOI:** 10.3390/foods12132553

**Published:** 2023-06-29

**Authors:** Aili Li, Jie Zheng, Xueting Han, Sijia Yang, Shihui Cheng, Jingwen Zhao, Wenjia Zhou, Yan Lu

**Affiliations:** 1 Key Laboratory of Dairy Science, Ministry of Education, College of Food Science, Northeast Agricultural University, 600 Changjiang Road, Harbin 150030, China; ailimail@neau.edu.cn (A.L.); s211001015@neau.edu.cn (J.Z.); s211001003@neau.edu.cn (X.H.); s221001003@neau.edu.cn (S.Y.); s221001007@neau.edu.cn (S.C.); s221001020@neau.edu.cn (J.Z.); s221002143@neau.edu.cn (W.Z.); 2 National Research Center of Dairy Engineering and Technology, Green Food Research Institute of Heilongjiang, Northeast Agricultural University, Harbin 150086, China

**Keywords:** lactose intolerance, dairy product production, lactase, fermentation, membrane separation, fortification, nutritional value, sensory properties

## Abstract

With increasing health awareness worldwide, lactose intolerance has become a major concern of consumers, creating new market opportunities for low-lactose/lactose-free dairy foods. In recent years, through innovating processes and technologies, dairy manufacturers have significantly improved the variety, and functional and sensory qualities of low-lactose and lactose-free dairy products. Based on this, this paper first covers the pathology and epidemiology of lactose intolerance and market trends. Then, we focus on current advantages and disadvantages of different lactose hydrolysis technologies and improvements in these technologies to enhance nutritional value, and functional, sensory, and quality properties of lactose-free dairy products. We found that more and more cutting-edge technologies are being applied to the production of lactose-free dairy products, and that these technologies greatly improve the quality and production efficiency of lactose-free dairy products. Hopefully, our review can provide a theoretical basis for the marketing expansion and consumption guidance for low-lactose/lactose-free dairy products.

## 1. Introduction

There is improving awareness of lactose intolerance in both research and dairy consumers. Generally, lactose intolerance is caused by primary lactose malabsorption. Lactase activity is highest at birth and declines after weaning. Undigested lactose is metabolized by intestinal microflora and converted into short-chain fatty acids (acetate, propionate, butyrate, lactate, and formate) and gases (hydrogen, methane, and carbon dioxide), causing gastrointestinal discomfort such as diarrhea, bloating and other symptoms. Long-term avoidance of dairy products due to lactose malabsorption or lactose intolerance may lead to malnutrition and skeletal disorders. Epidemiological surveys of lactose intolerance showed that approximately 70% of the world’s population was affected by lactose malabsorption, with prevalence as high as 95% to 100% in some Asian and African countries [1].

Current managements for lactose-intolerant people are mainly to replace regular dairy products with low-lactose and lactose-free products [2] and consume dairy products with exogenous lactase or probiotics. Low-lactose/lactose-free dairy products can reduce the incidence of gastrointestinal symptoms in lactose-intolerant patients while ensuring the intake of nutrients in milk. Sharp et al. [3] included 23 previous studies in their systematic review indicating that lactose-free milk and lactose hydrolyzed milk as substitutes for whole milk can reduce the risk of deficiencies and provide important nutrients for lactose-intolerant patients and healthy individuals. In addition, compared with other calcium-rich foods (such as vegetables and mineral water), low-lactose/lactose-free dairy products are a low-cost dietary source of calcium for lactose-intolerant patients [4]. Low-lactose/lactose-free dairy products are recognized as functional foods in the European Union [5]. An increasing variety of low-lactose/lactose-free dairy products, including liquid milk, Greek-style yogurt, kefir, cheese, ice cream and infant formula powder, have a growing appeal to consumers [6].

The requirements for developing low/lactose-free dairy products are avoiding lactose sources, ensuring the nutritional value and sensory properties of the product, and controlling production costs [7,8]. Generally, lactose content is required to be less than 1 g/100 g in low-lactose products and less than 10 mg/100 g in lactose-free products. The main ways to reduce lactose content [9] in dairy products include enzymatic hydrolysis, membrane separation, and fermentation. With the development of lactose removal technologies, the main challenge for manufacturers is to prepare low-lactose/lactose-free dairy products that are consistent with or exceed traditional dairy products in nutrition, flavor, and quality. Processing technologies and conditions are continuously being innovated, for example, studies reported characterization strains [10] of high yield and materials of immobilized lactase [11], coupling membrane, co-fermentation, etc. In this paper, the epidemiology and pathology of lactose intolerance are briefly introduced. Then, we focus on the market, processing technology, fortification, and improvement of low-lactose/lactose-free dairy products, hoping to provide theoretical reference for developing low-lactose/lactose-free dairy products suitable for lactose-intolerant patients.

## 2. Lactose Intolerance

### 2.1. Epidemiology

Lactose intolerance is a widespread worldwide problem. The ability of adults to digest lactose (lactase persistence) has become an important topic in genetics, medicine, and evolution. In recent years, the in-depth study of genetic mechanisms of lactase has provided a new theoretical basis for the epidemiological investigation and prevention of lactose intolerance. Storhaug, Fosse and Fadnes [1] presented a systematic review and meta-analysis of lactose intolerance by searching studies from Medline and Embase, including 62,910 participants from 89 countries. The global prevalence of lactose malabsorption estimated was 68%, and when using genotype data only, the estimate was 74%, whereas prevalence was 55% using lactose tolerance test data. Lactase gene polymorphisms have been widely used to predict lactose intolerance [12]. So far, there are 23 SNPs (single-nucleotide polymorphisms) known to be associated with lactase persistence. The most well-known and frequent SNP was identified at −13910 kb (rs4988235). LNP (lactase non-persistent) patients carry a C/C at this site, whereas LP (lactase-persistent) patients carry either a C/T or a T/T.

The distribution of LP genotypes shows significant national and regional differences, e.g., −13910C/T mostly occurrs in European and American countries. For northern Europeans, their endogenous lactase activity is still at a high level with increasing age. Anguita-Ruiz et al. [13] created an online interactive map of the distribution and frequency of LP genotypes worldwide. The prevalence of lactose intolerance increased from northern Europe to Africa and the Middle East and reached its highest in Asia. However, this trend was interrupted in countries with strong cultural admixtures, such as the US and countries in Europe. Chin et al. [14] reported −13910C/T in different ethnicities in the US. Only 17.9% of Caucasians were found to have the LNP genotype, while 96.5% and 69.2% of Asians and African Americans were found to have the LNP genotype, which indicated that there were great differences among different ethnicities within one country. In Africa and the Middle East, −13907:C>G (rs41525747), −14009:T>G (rs869051967), −13915:T>G (rs41380347) and −14010:G>C (rs145946881) were found to be more widespread [15,16]. Priehodová et al. [17] reported that the frequency of the LNP −13910*T variant was only 8.6% in nomadic Arabs, but 84.7% in non-nomads. Although increasing data are becoming available for lactase gene SNPs, studies of some geographical areas are relatively sparse, including Argentina, Uruguay, Cuba and Libya. Additionally, although Asia has a high prevalence of lactose intolerance, many well-known SNPs are difficult to detect, suggesting that there may be exclusive or new SNPs in this region [18]. For example, Peng et al. [19] found three new SNPs (−13838G/A, −13906T/A and −13908C/T) were associated with LNP in the Tibetan population. The creation of a broader database of lactase gene SNPs and collection of different SNP frequency data will be a direction of future analysis and research.

### 2.2. Lactase Gene and Pathology

**Figure 1:**
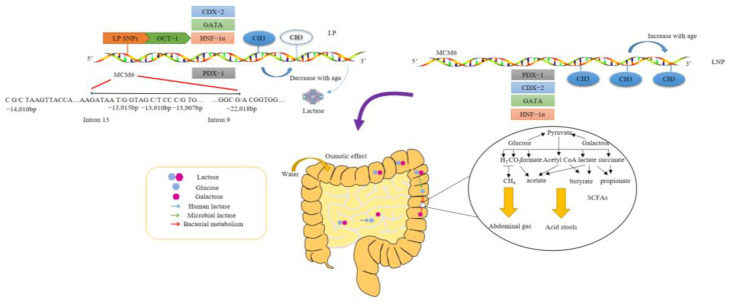
Mechanism of LP and LNP. LP: lactose persistence; LNP: lactose non-persistence; SNPs: single nucleotide polymorphisms; OCT−1: recombinant octamer binding transcription factor 1; PDX−1: pancreatic and duodenal homeobox 1; HNF−1α: hepatocyte nuclear factor 1 homeobox alpha; GATA factors were named after the consensus DNA-binding sequence (A/T)GATA(A/G), which is recognized by the zinc-finger domains common to all family members; CDX−2: caudal type homeobox 2; MCM6: minichromosome maintenance complex component 6; SCFAs: short-chain fatty acids.

It is now clear that lactase gene SNPs plays an important role in lactose metabolism (Figure 1). The MCM6 gene, which is 14 kb chromosomally upstream of lactase gene, contains a highly conserved promoter sequence. Some SNPs enhance transcription factors’ binding ability to lactase promoters by binding to them. The molecular mechanism for these SNPs is to create new binding sites for specific transcription factors, especially octamer-binding protein 1 (Oct-1). SNPs associated with LP can combine with transcription factors to promote the start of transcription, while SNPs associated with LNP cannot. Associated with LP, −13915*G was found to be able to interact with Oct-1 in vitro [20]. Other transcription factors involved in lactase activity regulation include caudal type homeobox 2 (Cdx-2), hepatocyte nuclear factor 1α (HNF1-α), and GATA-4, -5, and -6. Jensen et al. [21] found that the −14010*C variant associated with LP had greater binding affinity to Oct-1 than −14010*G. However, due to the stability of DNA sequences, transcriptional mechanisms cannot explain the programmatic decrease in lactase activity with age. The latest studies put forward that epigenetic mechanisms (mainly DNA methylation) could also be involved with LNP. Leseva et al. [22] found that a differential DNA methylation in the lactase gene through epigenome sequencing, which was closely related to activity of lactase and −13910C/T, was associated with the −13910C >T genotype. Moreover, the methylation level of this position combined with gene testing can predict lactase activity more accurately than the hydrogen breath test. LNP haplotypes containing the C(−13910) allele accumulated modified cytosines that silenced the regulatory elements in MCM6 and lactase gene, whereas the LP haplotype containing the T(−13910) allele displayed age-related modification changes that maintained lactase activity [23].

Lactase genotypes of hosts are known to influence the gut microbiome, and the influence seems to be modulated by lactose intake [24]. It is reckoned that small amounts of lactase are synthesized by the intestinal flora over a long period of genetic evolution in order to alleviate clinical symptoms of lactose intolerance. Among the colonic microbiota, association of lactase gene SNPs and the *Bifidobacterium* genus is widely identified [25]. Kurilshikov et al. [26] conducted a large-scale association analysis to identify host factors influencing human gut microbiome composition, and found the lactase gene locus reached study-wide significance and showed an age-dependent association with *Bifidobacterium* abundance. Goodrich et al. [27] reported that the association existed between *Bifidobacterium* and the lactase gene locus, and the direction of the genetic association showed lactase non-persisters harbored higher levels of *Bifidobacterium*. In addition, the latest study reported [28] that adults with lactose-tolerant genotype (GG) had higher milk intake, but lower *Bifidobacteria* compared to lactose-intolerant adults (AA/AG). There was a significant interaction between milk intake and LCT variant on *Bifidobacterium*, with a positive association between milk intake, and *Bifidobacterium* was observed only in lactose-intolerant but not in tolerant people.

Generally, lactose intolerance is considered to be a risk factor for reduced milk/calcium intake. According to a survey conducted by Cargill, 61 percent of US consumers avoided dairy products due to lactose intolerance or allergy to milk protein [29]. Obermayer-Pietsch et al. [30] detected lactase genotypes in 258 postmenopausal women and found that individuals with CC (LNP variant) had significantly lower milk calcium intake and decreased hip and lumbar spine bone density. However, recent studies have reassessed the association of lactase genotypes, dairy intake and bone health, and concluded that lactase gene SNPs had a weaker connection with bone health than anticipation. One study reported that LP and LNP genotypes were weak predictors of dairy intake [14]. Considering the geographical variation of lactase gene SNPs, Joslin et al. [31] evaluated the association of LNP and the heredity of numerous diseases in European populations, and found little evidence between LNP and reduction of bone mineral density. Hilliard et al. [32] explored the relationship between LNP and bone health in Africans, and the incidence of hip fracture and less consumption of animal protein was less correlated with LNP. Tolonen et al. [33] selected 882 Finnish women and 669 Finnish men as research objects. A slight difference in trabecular densities at the distal sites of radius and tibia was found in men between different lactase genotypes. Men with the T/T genotype were about 3% higher than those with T/C and C/C. No difference was found in women. More studies are needed to evaluate the association between lactose intolerance and bone health.

## 3. Lactose-Free Dairy Product Market

Lactose-intolerant consumers need nutritional products that they can utilize. It is known that 25% of consumers in India are motivated to buy or have started to buy lactose-free drinks. The lactose-free dairy products market in Vietnam is also booming, and relevant surveys showed that consumers preferred dairy products that are easy to digest [34]. The large-scale consumption of dairy products in China has just begun in the last 20 years, during which the concept of lactose intolerance has gradually become familiar to consumers. Up to 86.7 percent of Chinese adults are lactose-intolerant, which partly explains why China has the world’s largest market for plant-based protein beverages [35]. According to Szabo et al. [5], target group analysis in Hungary showed that currently two-thirds of lactose-sensitive consumers regularly consumed lactose-free products, most of which are female, lactose-intolerant, highly educated and aged under 30 years. Notably, lactose-free products are usually attractive to consumers with digestive problems because the products are always declared as easily digestible.

Lactose-free dairy products have become the fastest-growing part of the dairy industry. The compound annual growth rate of global lactose-free dairy products from 2017 to 2022 was about 7% and accounted for 80% of the total lactose-free products (sales of about 8.8 billion US dollars) [29]. In the US market, lactose-free milk accounted for 4.0% of the total liquid milk sold annually, and sales increased by 12% in 2017 [36]. Over the past five years, the range of lactose-free dairy products has continued to diversify, among which milk and yogurt were the most common lactose-free dairy products, while lactose-free butter, cheese and flavored milk are also on the rise. Świąder et al. [37] analyzed the market of lactose-free dairy products in Poland and categorized 75 lactose-free dairy products, including milk, yogurt, cream, quark, cheese and infant formula.

**Figure 2:**
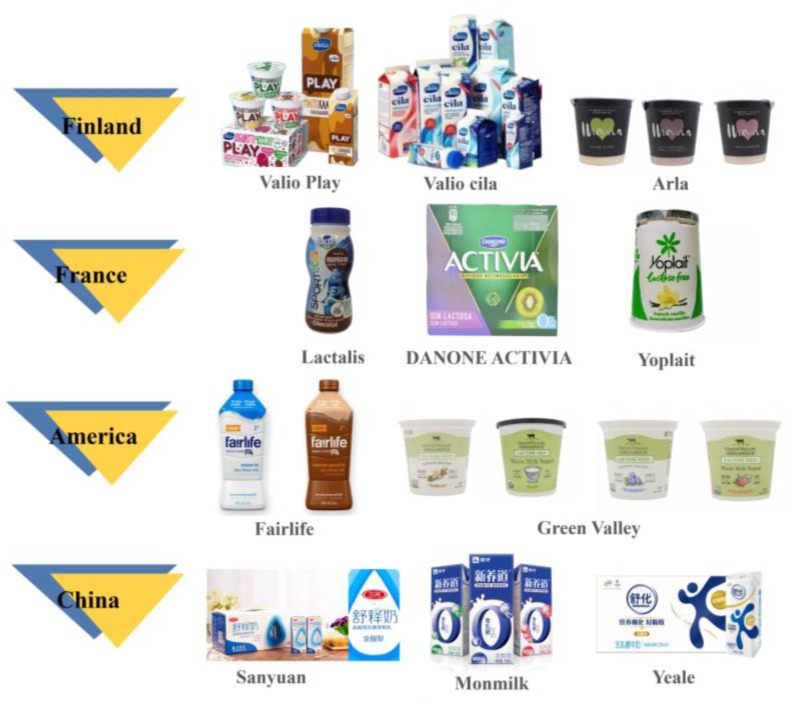
Representative lactose-free dairy brands of some countries.

Figure 2 shows representative lactose-free dairy brands of some countries. Finns have relatively high lactose intolerance prevalence in Europe, at 17 percent, leading to earlier popularity of lactose-free products. Valio, Finland’s largest food and dairy company, was the world’s first lactose-free dairy company, launching its first lactose-free milk in 2001. Lactalis of France was the world’s biggest cheese producer, and its products are low in lactose, often combined with low fat. Finland’s Arla Foods owned more than 30 brands, including the well-known Lactofree. Fairlife of America has launched a series of lactose-free ultrafiltered milk products, including shakes and recovery drinks, which are high in calcium and protein and have a strong position in the fast-growing lactose-free dairy market. As early as 2010, China produced only 300,000 t of lactose-free milk, accounting for 1% of liquid milk. In the past five years, while encouraging and guiding lactose-intolerant consumers to make reasonable choices, Chinese dairy enterprises have also increased production of lactose-free products. For example, Monmilk Xinyangdao was certified by the Lactose Intolerance Global Network (LIGN) in 2017, becoming China’s first lactose-free dairy brand certified by the international organization. These efforts are all leading lactose-free products to become more mainstream, standardized, and healthier.

## 4. Production of Lactose-Free Dairy Products

**Figure 3:**
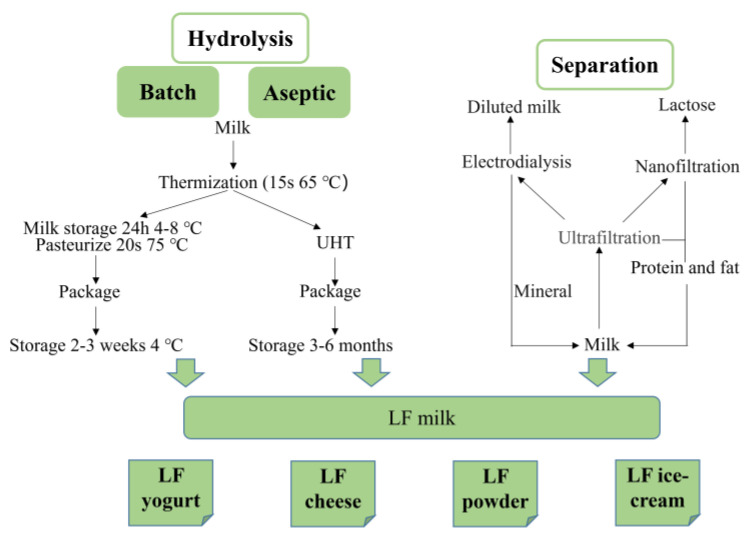
Production of lactose-free dairy products. LF: lactose-free.

The development of lactose-free and low-lactose dairy products made it possible for lactose-intolerant people to utilize the rich nutrients in milk, which is one of the effective ways to improve lactose malabsorption and lactase intolerance. Currently, there are three processes commonly used to reduce lactose content: enzymatic hydrolysis of lactose, membrane filtration, and fermentation. Studies have tried to combine several of above processes. Lactose-free milk can be further processed into lactose-free/low-lactose yogurt, cheese, milk powder, ice cream and other dairy products [29] (Figure 3). Many studies have confirmed that most consumers diagnosed with lactose intolerance can tolerate up to 10 g/day lactose and have no observable adverse reactions to 2 g/day lactose. Table 1 lists several regulations that mention lactose threshold. Although there are no common standards on allowable lactose threshold, in most countries, lactose content of low-lactose and lactose-free products are within 1 g/100 g and 10 mg/100 g, respectively.

**Table 1:** Regulations regarding lactose threshold in different countries have been issued by several authorities.

Threshold	Country/Region	Authorities	References
<1000 mg/L lactose as lactose-free	European countries	EFSA (European Food Safety Authority), 2010	-
<5000 mg/L lactose as lactose-free	China	EFSA (European Food Safety Authority), 2010,	[38]
<10,000 mg/L as low-lactose	India	FSSAI (Food Safety and Standards Authority of India), 2019	[39]
<100 mg/L as lactose-free	India	FSSR (Food Safety and Standards Regulation), 2019	-
0.1% (*w*/*w*) as lactose-free	Italy	Italian Health Ministry	[40]

### 4.1. Separation of Lactose

Among methods of separating lactose, the most well-known and widely used are methods of membrane separation, including ultrafiltration, nanofiltration, reverse osmosis, and electrodialysis. In different fields of the dairy industry, membranes are applied to shelf life extension of milk, whey processing, cheese industry, milk protein processing, fractionation of milk fat and desalting or demineralization [41]. The key problem in the removal of lactose from milk is the separation of proteins and lactose, and the broad particle size distribution of proteins in milk reduces the separation efficiency of proteins and lactose [42]. Protein and fat are blocked, while lactose and small molecules are allowed to pass through in ultrafiltration and nanofiltration. Polymer ultrafiltration membranes are usually used in industrial practice because of their ease of preparation and cost-effectiveness. By screening and comparing, Sanchez-Moya et al. [43] found GR60PP (a polysulfone ultrafiltration membrane) to be the most efficient membrane, as 90% of the lactose was separated and 100% protein was recovered. This indicated that significant adsorptive fouling and pore blocking were the main causes of high rejection of lactose. Researchers focused on reducing membrane fouling mainly caused by protein. Attempts were made to achieve high permeation flux and high separation efficiency through ultrasound assistance, optimization of operating conditions, and development of cleaned membranes. Use of ultrasound under optimal power was demonstrated to be able to reduce the fouling by 32% [44]. Sofuwani, Aslina, and Mazlina [45] found the lowest lactose rejection (77.71%) was achieved when adapting 5 kDa cross-flow hollow fiber ultrafiltration membrane and operation parameters of 0.55 bar trans-membrane pressure and 0.74 L min^−1^ feed flow rate. Graphene oxide membrane allowed lactose to diffuse through the nanochannel and exhibit higher lactose permeation flux (2.87 kg m^−2^ day^−1^) than commercial nanofiltration (0.57 kg m^−2^ day^−1^) and ultrafiltration (1.61 kg m^−2^ day^−1^). In addition, the fouling layers on graphene oxide membrane were porous, facilitating higher permeation flux and water flux recovery. Graphene oxide membrane is very promising for lactose separation of lactose-free milk [46].

Generally, ultrafiltration membranes have low permeability and high protein rejection, whereas microfiltration membranes have higher permeability, but higher protein losses [19]. Another common practice in dairy processing is called diafiltration, during which water is added to the concentrates to increase the flux and continue the separation. The trade-off between permeability and selectivity reduces the separation efficiency of proteins and lactose [20]. A microfiltration membrane with smaller pores may be a good choice. Qi et al. [42] selected ceramic microfiltration membranes with high permeation flux and high rejection performance and prepared low-lactose milk with a lactose concentration of less than 5 g/L. The molecular weights of mineral and lactose are very close, so it is difficult to separate them by a single membrane. Coupling membranes are superior, as large molecules such as proteins and fats are first retained using ultrafiltration. Minerals are intercepted by electrodialysis and lactose is recovered by nanofiltration. For example, Zhang et al. [47] obtained low-lactose milk powder with lactose concentration of less than 0.2% and recovered high-purity lactose powder (95.7% lactose content) as a byproduct. Further, membrane separation can be combined with hydrolysis and enzymatic membrane bioreactor function by optimizing GOS production [48].

Several studies explored methods to separate lactose by chromatography and freeze concentration. For example, lactose-free milk produced by Valio Ltd. (Helsinki, Finland) employed chromatography, membrane separation and enzymatic hydrolysis. Freeze concentration is a widely accepted technology and is able to preserve thermosensitive food with high nutritional value. In this process, the temperature of food liquid decreases below its freezing point, and the concentration of liquid is reached by removing water in the form of ice crystals. A new type of lactose-free dairy product was developed by combining progressive freeze concentration with vacuum-assisted block freeze concentration, because carbohydrates accumulated more during progressive freeze concentration, while in block freeze concentration, protein is more likely to enter concentrated liquid [49]. Batista et al. [50] aimed to develop a new system for lactose removal through bioaffinity chromatography. In the research, brosimin, a lactose-binding lectin, was first extracted from *Brosimum gaudichaudii* and immobilized onto polyaniline. The system removed 47% of lactose from skim bovine milk.

### 4.2. Enzymatic Hydrolysis of Lactose

The process of lactose hydrolysis does minimal damage to the nutritional components in milk and is highly specific. Two processes, batch and aseptic, are used in producing lactose-free milk. In the batch process, neutral lactase is added to milk under slow stirring until lactose is fully hydrolyzed, after which the milk is pasteurized, homogenized, and packaged. The batch process lacks pasteurization during the hydrolysis phase, and enzyme dosage is relatively high because the reaction occurs at low temperatures to prevent microbial spoilage [29]. Research on cold-active β-galactosidase has improved this defect. In the aseptic process, milk is first sterilized using the UHT procedure, after which sterile lactase is injected into the milk just before packaging. Although the lactase dosage decreases compared to the batch process, the aseptic process requires special equipment, and process control is absent since the hydrolysis reaction continues after packaging.

#### 4.2.1. Microorganism Source of Lactase

The most plentiful resource of β-galactosidase (also known as lactase) are microorganisms, which have advantages of a short production cycle and high production yield. Research has been conducted on the optimization of reaction conditions, purification and characterization of β-galactosidase. Commercially available β-galactosidase mainly includes *Aspergillus oryzae*, *Aspergillus niger*, *Escherichia coli*, *Kluyveromyces lactis* and *Kluyveromyces fragilis* [51]. β-galactosidases from microorganisms have different enzymatic characteristics and structure. For example, *Kluyveromyces lactis*, the most reported microorganism in the literature, showed high hydrolytic performance that was relevant with an exclusive insertion in loop 420–443 of its catalytic site, which enhanced affinity of lactase to lactose [52].

Isolation and characterization of β-galactosidase that fulfill the demands of production of low-lactose dairy products remains a hot topic. Genetic engineering technology was used to express the lactase with good enzymatic characteristics in heterologous expression (*E*. *coli*, *Lactococcus* and *Pichia pastoris*, et al.). As presented in Table 2, studies showed that the production of recombinant β-galactosidase of different microbial sources increased. Generally, commercial β-galactosidases feature the high lactose affinity (K_M_) and low product inhibition (K_I_) by galactose, which is adverse for hydrolysis reaction. Engineering technology is used to get satisfactory lactose affinity (K_M_) and product inhibition (K_I_) [53]. The optimal pH and temperature of commercial β-galactosidases are 7.0 and 35 to 40 °C, which are susceptible to result in the contamination of milk. Thermostable and cold-adapted β-galactosidases had significant advantages in processing, such as higher substrate solubility and reaction rate, as well as lower probability of microbial contamination [54]. Furthermore, lysis of cells and extraction of intracellular lactase increased the costs of production. Ren et al. [55] used the twin-arginine (Tat) signal peptide PhoD to direct the secretion of the β-galactosidase, which is a new pathway to improve the secretion amount of lactase. It provided a new way to improve lactase production.

Galactose oligosaccharides (GOSs) can be applied as prebiotics in a variety of dairy products. The global prebiotics market size exceeded US$ 2.90 billion in 2015, with an expected growth of about 12.7% and a profit of about $10.55 billion by 2025 [56]. Transglycosylation is another property of β-galactosidase. GOSs are synthesized during this process, which reduces concentration of lactose and increases the value of low-lactose milk. In conclusion, the characteristics of lactase are important for the enzymatic production of lactose-free dairy products. The dairy industry aims to produce standard lactose-free milk with low cost and high added value.

**Table 2:** Studies on strain producing lactase of high activity.

	Enzyme Source	Process	Advantages	References
Strains resistant to low/high temperature and acid environment	*Alteromonas* sp.ML117	*Alteromonas* sp. ML117. β-galactosidases were heterologously expressed in *E. coli* and the recombinant lactase was purified.	Recombinant β-galactosidase was a cold-adapted variant and hydrolyzed 86% lactose of milk after 24 h at 10 °C. The enzyme is NaCl-tolerate.	[57]
	*Picrophilus torridus* DSM 16176	The enzyme was purified 110-fold and determined.	This enzyme is thermostable. At 70 °C, it retained 76% and 42% activity after 30 and 120 min.	[58]
	*Anoxybacillus* sp.AH1	The enzyme was purified 10.2-fold.	The purified enzyme was highly stable and retained at 71% of the original activity at 60 °C and 53% at 70 °C within 120 min.	[59]
	*Aspergillus* niger van Tiegh	Extracellular β-galactosidase was purified to homogeneity using a combination of gel filtration, ion-exchange, chromatography.	The enzyme is highly stable when exposed to simulated gastric conditions in vitro. It retained 68% of original activity. Activity of capsule is some 3.5-fold more than commercial enzyme.	[60]
Strains with lactose affinity and reduction of product inhibition	*Bifidobacterium adolescentis*	β-galactosidase gene found in *Bifidobacterium adolescentis* and was expressed in *E. coli*.	This enzyme had a Km of 3.7 mM. It exhibited low product inhibition by galactose with a Ki of 116 mM and high tolerance for glucose.	[61]
	*Aspergillus candidus*	Four amino acid positions (Tyr96, Asn140, Glu142, and Tyr364) were selected for mutation based on their molecular bindings with galactose using site-directed mutagenesis.	β-galactosidase Y364F (Tyr364 mutant) had a galactose inhibition constant (K_I_) of 282 mM, which is 15.7-fold greater than that of the wild-type enzyme.	[62]
Strains with high transgalactosylation capacity	*Klebsiella oxytoca* ZJUH1705	Two β-galactosidase genes were isolated from a novel β-galactosidase-producing *Klebsiella oxytoca* ZJUH1705. Two β-galactosidase genes were cloned, expressed in *E. coli* and purified.	β-gal 2 had a high trans-glycosylation capacity. Adding β-gal 2 in lactose with the ratio of 2.5 U/g, a high GOS yield of 45.5%was obtained.	[63]
	*Bacillus* sp. D1. BglD1	A novel β-glucosidase, BglD1 was screened and cloned from the deep-sea bacterium. a mutant BglD1:E224T was generated based on the semi-rational design.	BglD1 hydrolyzed 88.5% lactose and produced 3.3 g/L GOS when using milk as the substrate. The GOS yield of its mutant was 11.5% higher than that of BglD1.	[64]
	*Paenibacillus barengoltzii*	β-galactosidase gene was cloned, expressed in *E. coli* and purified.	The recombinant β-galactosidase exhibited high trans-glycosylation activity. Maximum yield of GOS was 47.9% at a lactose concentration of 350 g/L.	[65]
	*Alteromonas* sp. ANT48	β-galactosidase gene was cloned, expressed in *E. coli*.	90.6% of the lactose was hydrolyzed at 40 °C within 15 min. GOS yield reached 30.9%.	[66]
	*Streptococcus thermophilus*	Site-directed mutation strategy was attempted to genetically modify β-galactosidase (the enzyme and its mutant were named BagQ and BgaQ-8012 respectively)	The GOS yields increased to 5.8 and 8.3 g/L adding BgaQ or BgaQ-8012. Addition of the β-galactosidases reduced lactose content by 49.3% and 54.4% respectively in yogurt.	[67]

#### 4.2.2. Immobilized Lactase

Immobilization is widely applied in production of lactose-free products because of operational stability, reusability and easy recovery of β-galactosidase in continuous process. Absence of the enzyme in the final product increases its stability. Retained activity of β-galactosidase after recyclable use is critical when deciding whether the immobilized enzyme is suitable for manufacturing. The performance of lactase is greatly influenced by the sources and purity of the enzyme, as well as the type of immobilization method and support materials used. Immobilization methods include adsorption, covalent binding, cross-linking, encapsulation and entrapment. Support materials significantly affect properties (thermal resistance, chemical resistance, mechanical properties and biocompatibility) of immobilized enzymes [68] (Table 3). Classic support materials for β-galactosidase immobilization include alginate, chitosan, silica, resin and so on. New materials are continuing to develop and the particle size of these materials are generally at the nanoscale, which provides large surface-volume ratio, high surface reaction activity and high catalytic efficiency. Nanomaterials mainly applied to the immobilization of β-galactosidase are carbon nanotubes, silicon dioxide nanoparticles, nanodiamonds, silver nanoparticles and zinc oxide nanoparticles [69]. Furthermore, new materials (such as graphene oxide, mesoporous and electrospinning material) are abundant, with a lot of functional groups. For example, many carboxylic (COOH), hydroxyl (–OH) exist in the surface of graphene oxide, which facilitates enzyme–matrix interactions and make it easy for β-galactosidase to be modified [70]. The future direction of development and application lies in reducing costs, sustaining activity of immobilized enzymes, and synthesis of new support materials.

### 4.3. Fermentation

Fermentation hydrolyzes 20–30% of the lactose of milk, and protein and fat into peptides, amino acids, fatty acids, which makes it easy for human to digest and absorb nutrients. Traditional yogurt is fermented by *Lactobacillus bulgaricus* and *Streptococcus thermophilus*. The lactose content of the fermented milk decreased and ranged between 4.6% and 3.7%. Moreover, the yogurt strains produce few β-galactolactases, which further hydrolyze lactose. A systematic review by Savaiano et al. [79] concluded that there was a positive correlation between yogurt consumption, improved lactose digestion and improved lactose tolerance symptoms. Greek-style yogurt has become popular in the United States since its first appearance in 2007 and gained 50% market share of yogurt quickly. Greek-style yogurt is made on the basis of yogurt after concentration, centrifugation, isolation of whey or supplementation of protein to achieve the thick and creamy texture [80]. Greek-style yogurt has always been claimed to have high protein (8–12%), low lactose (reduced by half) and low fat. Kefir is another common fermented dairy product. Lactic acid bacteria and yeast in kefir constitutes a complex symbiotic relationship, which is responsible for alcohol and lactic acid fermentation, respectively, giving the product unique flavor [81] and producing functional substances (bioactive peptides, cellular polysaccharides and amino acids, etc.) [82]. The lactose content of kefir is 3.1 g/100 g, and its unique microorganism helps to relieve lactose intolerance by regulating and maintaining the balance of intestinal flora [83]. Cheese is a natural low-lactose dairy product. Most lactose will be excreted with whey during the processing and preparation of cheese. For hard and semihard cheese, the remaining lactose continues to be converted into lactic acid during the cheese ripening process, making them natural lactose-free products. Panseri et al. [84] detected carbohydrates in lactose-free dairy products, and the concentration of lactose in PDO hard cheese was below 0.0001 mg/kg. There are a wide variety of low-lactose/lactose-free cheeses available on the market for lactose-intolerant consumers. The lactose content of Asiago PDO, Gorgonzola PDO, Emmentaler PDO, Pecorino Toscano PDO, Piave PDO, Stelvio PDO, and Montasio PDO ranges from 10 mg/kg to 100–1000 mg/kg [40]. The lactose content of Grana Padano PDO, Parmigiano Reggiano PDO, and Pecorino Romano PDO is less than 10 mg/kg.

The lactose content of traditional fermented dairy products still does not meet the needs of lactose-intolerant people. Lactase can be added to further hydrolyze lactose before (pre-hydrolysis) or during (co-hydrolysis) fermentation. These two hydrolysis methods will affect the fermentation characteristics of yogurt to some extent. Generally, lactose is hydrolyzed into glucose and galactose by lactase, and lactic acid bacteria can directly utilize glucose to produce lactic acid, thus shortening the fermentation time. In the meantime, lactic acid bacteria produce more exopolysaccharides and possibly result in higher viscosity. During storage, a large number of flavor substances such as acetaldehyde and 2,3-butanedione were synthesized at higher levels in low-lactose yogurt than in regular yogurt. For example, Yamamoto et al. [85] found that, compared with unhydrolyzed milk, *Lactobacillus bulgaricus*, extracellular polysaccharide synthesis and viscosity of pre-hydrolyzed milk significantly increased, which may be related to the decrease in dissolved oxygen and the increase in formic acid concentration caused by utilization of glucose. Martins et al. [86] found that the processing time of yogurt co-fermented with *Bifidobacterium animalis*, *Lactobacillus acidophilus* and typical microorganisms of yogurt and co-hydrolyzed reduced from 4.55 h to 3.68 h. Lactose conversion increased from 15.2% to 97.9%.

Pre-hydrolysis to control the lactose content in the final product relatively easily. For example, Raza et al. [87] produced low-lactose cheese containing prebiotics (lactose content 0.8 g/100 g) by converting lactose into galacto-oligosaccharide by adding exogenous lactase. The appearance and overall acceptability of this product are similar to control cheese. Rutkowska et al. [88] added commercial lactase to pre-hydrolyze for 24 h and then added fermentation culture to produce lactose-free kefir. The results showed that the lactose content of the product was as low as 0.1 g/100 g and contained double the ketones (especially 3-hydroxy-2-butanone and 2,3-butanedione), which probably contributed to the high intensity of creamy aroma. Lactose-free kefir is sweeter in taste than traditional kefir and is favored by elderly consumers. The content of lactose in co-hydrolyzed yogurt cannot meet the demand for lactose-free products. Most commercial neutral lactases are completely inactivated when pH is <5.5, and the acidity of yogurt reaches this threshold after 2–3 h of fermentation, which can be solved by adding excess lactase or acidic lactase. However, compared with pre-hydrolysis, co-hydrolysis protects the activity of the fermentation strains and preserves the flavor of fermented milk. Some bacteria may not adapt to the change in the main carbon source from lactose to glucose and the increase in osmotic pressure of milk after rapid hydrolysis of lactose during pre-hydrolysis, which inhibits the activity of lactic acid bacteria to a certain extent. Popescu et al. [89] compared the effects of unhydrolyzed, pre-hydrolyzed and co-hydrolyzed on sensory characteristics of yogurt, and the results showed that the co-hydrolyzed yogurt had the best flavor, which was speculated to be related to the production of more aromatic compounds. Ibrahim et al. [90] evaluated the effects of pre-hydrolysis and co-hydrolysis on the sensory and physicochemical properties of fermented camel milk, and the results showed that the number of bacteria of both the two were higher than unhydrolyzed milk. The decreasing speed of pH and increase in apparent viscosity of hydrolyzed milk was significant, and the co-hydrolyzed camel milk had the best sensory scores. In addition, starter culture highly affected the fermentation characteristics of dairy products. Schmidt et al. [91] researched the changes in the rheological properties of yogurt under pre-hydrolysis and co-hydrolysis conditions, and the results showed that the apparent viscosity of yogurt was affected more by the starter strains than by the hydrolysis method.

## 5. Detection and Determination of Lactose

Traditionally, detection of lactose in dairy products is carried out using different methods, such as gravimetry, polarimetry, enzymatic methods and high-performance liquid chromatography (HPLC). These methods have been proven to perform well in the un-hydrolyzed milk system. However, when it comes to low-lactose/lactose-free dairy products, they suffer from various drawbacks [39]. The formation of other saccharides during hydrolysis and such low concentration of lactose hinder lactose determination in lactose-free dairy products. There is also a requirement for methods with high sensitivity and precision. Currently available methods are basically improved by enzymatic methods and chromatography. Enzymatic kits and biosensors are based on enzymatic reactions and are used frequently at the commercial level because they are fast and easy to use. Mangan et al. [92] described a novel enzymatic low-lactose determination method, which is based on an optimized glucose removal pre-treatment step followed by a sequential enzymatic assay. Sensitivity was improved through the extension of the typical glucose detection biochemical pathway to amplify the signal response. The limit of detection (LOD) and limit of quantification (LOQ) of this method are 0.13 mg/100 g and 0.44 mg/100 g, respectively.

Chromatography includes a wide range of improved and coupled methods, such as gas chromatography (GC) [93], ultrahigh-performance chromatography coupled with tandem mass spectrometry (UHPLC-MS/MS) [94], high-performance anion-exchange chromatography with pulsed amperometric detection (HPAEC-PAD), high-performance thin-layer chromatography coupled with a fluorescence detector (HPTLC-FLD) [95] and so on. Among all the methods, HPAEC-PAD shows the highest accuracy and specificity without any interference from other disaccharides. Monti et al. [96] reported the obtained LOD and LOQ values were, respectively, 0.25 and 0.41 mg/100 g for lactose, 0.14 and 0.27 mg/100 g for galactose, and 0.16 and 0.26 mg/100 g for glucose in Grana Padano PDO cheese. However, it is quite expensive and therefore not used widely.

Other methods include nuclear magnetic resonance (NMR) [97], capillary zone electrophoresis [98] and depression in freezing point [99]. These methods are applied for the determination of lactose in milk, but still very limited in lactose-free dairy products.

## 6. Fortification of Lactose-Free Dairy Products

### 6.1. Function

More and more clinical studies report that probiotics can assist in relieving lactose intolerance [100,101,102]. Although the specific mechanism is not clear, it is generally believed that changes in microbial flora composition by probiotics and enhancement of immune function are the basis of their effects [103]. Fermented milk is one of the most ideal matrices in the culture of probiotics. *Lactobacillus* (including *Lactobacillus acidophilus*, *Lactobacillus rhamnosus*, *Lactobacillus casei*, etc.), *Bifidobacterium* and *Saccharomyces* and other probiotics are often used in the production and preparation of fermented milk. A systematic review by Oak et al. [104] evaluated the efficacy of eight probiotics in the treatment of lactose intolerance, and the results showed that although the efficacy of different strains in improving intestinal digestion was different, probiotics were generally positively correlated with the alleviation of lactose intolerance. A randomized, double-blind, crossover study by Vitellio et al. [105] showed that formulations of *Bifidobacterium longum* BB536, *Lactobacillus rhamnosus* HN001, and vitamin B6 together significantly improved bloating and constipation in lactose-intolerant patients. Compared with placebo, probiotics drove the enrichment of bacteria involved in lactose digestion in the gut and also produced a few lactases to promote the hydrolysis of lactose in the human body.

Prebiotics are also functionally beneficial for gut health. However, compared with probiotics, there are few studies on improving lactose intolerance using prebiotics alone [106]. At present, only RP-G28 (a galacto-oligosaccharide of more than 95% purity) has been reported. Savaiano et al. [107] reported RP-G28 or placebo was administered to 85 patients with lactose intolerance for 35 days and RP-G28 subjects were six times more likely to claim lactose tolerance post-treatment. Similarly, a randomized, double-blind, placebo-controlled clinical trial by Chey et al. [108] also found that 30 days of treatment with RP-G28 reduced abdominal pain by 50% in lactose-intolerant patients, which was six times more effective than placebo. Presumably, selective utilization of prebiotics by host microorganisms increased the survival of probiotics in the gastrointestinal tract. That is why more studies are focusing on the combination of probiotics and prebiotics. Commonly used prebiotics include galacto-oligosaccharide, fructooligosaccharide (FOS), inulin, etc. For example, Pereira et al. [109] used *Lactobacillus acidophilus* LA-5, *Bifidobacterium lactis* Bb-12 and inulin to produce fermented milk with lactose below 0.1%, and this product had a high sensory score and probiotic activity. Table 4 lists some studies of lactose-free fermented dairy products supplemented with probiotics/prebiotics. The focus of these articles was ensuring the viability of probiotics in the products (more than 10^6^ CFU/g at least) to promote lactose digestion and absorption.

### 6.2. Nutrition

Milk is a good source of calcium, vitamin B2, vitamin A, and vitamin D. Consumption of 250 mL milk provides 26–40%, 23–52%, 10–24% and about 5% of mineral and vitamins US RDA (recommended dietary allowance) recommendations, respectively. However, lactose-intolerant people often avoid eating dairy products. It is also difficult for them to effectively utilize the rich minerals, vitamins and other nutrients in milk, thus resulting in osteoporosis and other adverse health consequences. Although human clinical trials cannot confirm the effect of lactose on enhancing calcium bioavailability [116], there are numerous animal studies supporting lactose as an enhancer of calcium absorption. Lactose-free products should be fortified to contain at least 20% more calcium than the recommended calcium intake. Lactose-free dairy products should differ from skim dairy products only in lactose content, and can basically meet the nutritional needs of lactose-intolerant patients and other consumers. Within the limits of standard regulations, lactose-free products can be fortified with additional nutrients to satisfy the specific physiological needs of lactose-intolerant individuals. In the US and Canada, milk is mandatorily fortified and is an important source of vitamin D. Vitamin D food fortification policy, started in 2003, recommends that all liquid dairy products, lactose-free milk-, soy-, and cereal-based drinks are to be fortified at a concentration of 0.5 μg/100 g. Jaaskelainen et al. [117] analyzed the health survey data of 6134 and 4051 Finnish adults from 2000 and 2011, and found that the average serum 25-hydroxyvitamin D (S-25(OH)D) concentration increased from 48 nmol/L to 65 nmol/L. This increase is mainly explained by food fortification, especially of fluid milk products.

In addition, many lactose-intolerant consumers turn to plant-based alternatives after finding it hard to digest dairy products. Many plant-based beverages have even higher levels of fortified calcium than dairy products. A review compared nutrient density in milk and 17 plant-based beverages, with milk containing 120 mg/100 mL calcium and fortified plant-based beverages containing 42–197 mg/100 mL calcium [118]. However, the bioavailability of calcium supplements in plant-based beverages was inferior to that in cow’s milk, where the absorption of calcium triphosphate was only 75% of calcium in milk. According to Heaney et al. [119], calcium precipitates can also occur in plant-based beverages. The average calcium content of calcium-fortified soy drinks after shaking is only 59% of what is claimed on the label, while the average calcium content of unshaken soy drinks is only 31%.

Apart from direct addition of calcium, phosphorus, and vitamin D, there are other ways to increase the nutritional value of lactose-free dairy products. For example, Dantas et al. [120] increased the carbohydrate and protein content of lactose-free milk by 2.95 and 3.00 times, respectively, through a freezing concentration process. da Silva et al. [121] replaced milk powder with lactose-free whey protein concentrate, which increased the protein and calcium content of Greek yogurt while improving the rheological and sensory properties of the product.

## 7. Improvement in Sensory Properties and Quality of Lactose-Free Dairy Products

Since consumers are easily driven by the senses, lactose-free dairy products manufacturers attached great importance to sensory properties. Lactose-free dairy products similar to skim milk are believed to be more popular. However, commercially available lactose-free milk typically utilizes lactase to hydrolyze lactose into galactose and glucose, so it tends to taste sweeter than regular milk. Commercial lactase can also have proteolytic activity that releases peptides and free amino acids generating nonenzymic browning and “off” flavors during the shelf life. The extent of proteolysis and product deterioration identified depended on the lactase preparation used and its purity [122]. However, research on the influence of microbial sources on proteolytic activity is very limited. Nielsen et al. [123] compared five commercial lactase preparations from different companies and of different purity. Among them, lactase preparations with the lowest purification gave rise to the highest degree of proteolysis and aggregation. Thus, lactose-free milk was more susceptible to Maillard reaction, and storage increased hexosylation up to elevenfold in lactose-free UHT milk [124]. Lactose-free milk is also often described as having a cooked and eggy flavor. A recent study also showed that the volatile sulfur compounds that create eggy flavor in milk result from Maillard reactions between reducing sugars (lactose) and cysteine and methionine amino acids [125]. When lactose-free milk is produced by a batch process, the lactase and its proteolytic activity disappear after heat treatment. In contrast, under an aseptic process, lactase remain active throughout the shelf life, which could damage the quality of the product during storage. Tossavainen O et al. [126] found that both batch and aseptic processes resulted in proteolytic hydrolysis compared to UHT milk without lactose hydrolysis. However, when the storage temperature was below 5 °C, the proteolytic activity was inhibited. Therefore, the use of commercial lactase preparations with high purity and strict control of storage temperature can ensure the quality of lactose-free milk during the shelf life.

It is generally believed that fermentation can not only improve the flavor of products but also increase functional substances or the content of nutrients in products [127]. The sensory evaluation of many lactose-free yogurts is better than that of unhydrolyzed yogurt. On one hand, this may be because lactic acid bacteria can directly use glucose to produce more aromatic compounds, alcohols, esters, aldehydes, ketones and other flavor substances. On the other hand, more exopolysaccharides are produced and increase the apparent viscosity and improve dehydration characteristics. In addition, some additives can also improve the quality of lactose-free fermented products. For example, Moreira et al. [128] prepared a low-lactose yogurt with added fiber (*Ceratonia siliqua* L.) with content of 1.16–1.44 g/100 g, which had good acceptability. Synthesis of GOS using lactase with high trans-glycosylation activity is another common method to increase the added value and improve the flavor of dairy products. For example, Raza et al. [87] produced prebiotic-enriched cheese using lactase from *Kluveromyces lactis*. Lactose content decreased to 56.25%. The prebiotic cheese has a similar appearance and overall acceptability, as does the control cheese, except for taste and texture, which were improved by trans-glycosylation.

Hydrolyzed milk can further produce lactose-free milk powder, but there are still many problems in the production of lactose-free milk powder. Lactose-free milk powder is more hygroscopic, which is related to the fact that there are more molecules in the amorphous state (glucose and galactose) during drying. Shrestha et al. [129] showed that spray-drying of skim milk with hydrolyzed lactose resulted in very low cyclone recovery of 25% and a large amount of powder remained stuck inside the spray dryer. Torres et al. [130] found that with the increase in lactose hydrolysis rate, adhesion to the drying chamber also increased, due to higher levels of particle agglomeration. In addition, due to the increase of reducing sugar in hydrolyzed milk powder, nonenzymatic browning in high-temperature processing occurs easily, resulting in increased Maillard reaction products and odor. For example, Queiroz et al. [131] found that lactose hydrolysis of goat’s milk resulted in greater darkening and increased free fat content. Naranjo et al. [132] researched the kinetics of Maillard reaction in lactose-hydrolyzed milk powder and found that hydrolyzed milk powder was prone to protein deterioration, mainly because galactose reacted faster with lysine than lactose in dairy products. At the same time, temperature is the most important factor. Lower temperatures can reduce the deterioration of nutrients during storage.

In the production of lactose-free ice cream, since the solubility of lactose at room temperature is only 20% of that of sucrose, there are difficulties, including crystallization of α-lactose during freezing and formation of sandy texture. Another problem in lactose-free ice cream production is the low sweetness of lactose, which is about 20% of sucrose. Membrane was combined with enzymatic method to solve the problem of the high sweetness of lactose-free milk [133]. Furthermore, when preparing lactose-free ice cream, the addition of lactase can be used as a sugar-reduction method, because hydrolysis of 70% of the lactose in milk increased the sweetness of milk or yogurt to the same degree as adding 2% sugar [134]. For example, Abbasi et al. [135] showed that with the increase in lactose hydrolysis, the apparent viscosity increased and freezing point decreased, and the sensory properties of ice cream hydrolyzed with 75% lactose were similar to those of ice cream with 25% sugar reduction.

## 8. Conclusions

With the awakening of consumer health awareness, lactose-intolerant people tend to choose healthier and safer lactose-free dairy products. Dairy enterprises and manufacturers have improved processes and technologies, using immobilized enzymes, genetic engineering, membrane filtration, and fermentation to remove lactose. Different methods have their own advantages and disadvantages. The right choices and combination of several methods can improve sensory properties, decrease the cost of production, and increase the nutritional value and functional effect under the premise of low lactose content. Research in the future will focus on the improvement in production and development of lactose-free dairy products of high quality, so as to provide more choices for lactose-intolerant patients.

## Figures and Tables

**Table 3:** Application of immobilized lactase in preparation of low-lactose milk.

Method	Support Material	Activity of Enzyme and Ability of Hydrolysis	Other Advantages	References
Covalent binding	Eupergit CM	The activity of immobilized enzyme decreased after 20 times of repeated use, and reached 99.3% after 15 days of storage. Lactose was completely hydrolyzed within 4 h.	Storage stability and activity of enzyme increase.	[71]
Cross-linking and adsorption	Modified arabic gum-based hydrogel	After 3 cycles, activity of immobilized β-D-galactosidase was 52.79% of the initial enzyme.	Improve the efficiency of lactose hydrolysis and lower costs.	[72]
Adsorption	Fe-chelated cryogel disk	The immobilized lactase lost 29.2% after 70 days and preserved 64.9% of initial activity after 25-runs.	The optimum temperature of immobilized lactase increase.	[73]
Covalent binding	Mesoporous silica/titania with a chitosan coating	Lactase retained approximately 90% of initial activity and achieved full conversion of lactose even after 15 cycles in batch system.	Enzyme is hard to deform and demonstrates high operational stability for application and manufacturing.	[74]
Entrapment	Bacterial cellulose nano crystal	β-galactosidase retained 80% activity after 12 cycles of use.	β-galactosidase showed higher stability to various range of pH and temperature.	[75]
Covalent binding	Gluconic acid coated fullerenes	β-galactosidase was able to be recovered easily and retained 89% activity after 6 repeated uses.	Obvious improvement in lactose hydrolysis was observed at high temperature.	[76]
Entrapment and adsorption	Halloysite nanotubes and cellulose nanocrystals	Enzyme retained 76% activity after 12 cycles.	Enzyme was more thermostable at 55 °C than the free enzyme.	[77]
Covalent binding	Modified gold nanoparticles	β-galactosidase exhibited greater operational activity after 6 reuses.	Stability was significantly enhanced at wider temperature, pH and higher galactose concentrations.	[78]

**Table 4:** Lactose-free fermented dairy product supplemented with probiotics/prebiotics.

Product	Study	Conclusion	References
Low-lactose fermented goat milk	Development of low-lactose fermented goat milks with *Bifidobacterium animalis* ssp. lactis Bb-12 and evaluate the effect of prior lactose hydrolysis on the viability of *Bifidobacterium animalis* ssp lactis Bb-12.	The lactose hydrolysis of milk resulted a higher hardness in probiotic fermented goat milk. Moreover, the lactose-free probiotic fermented milk had a more distinct sweet taste than the control one and was characterized by a less sour flavor.	[110]
Lactose-free functional yogurt	Physicochemical, rheological, and microbiological properties of lactose-free functional yogurt supplemented with FOS.	Lactose hydrolysis and FOS supplementation increased acidification rate during fermentation of yogurts. FOS helped to improve syneresis.	[111]
Concentrated lactose-free yogurt	Effect of encapsulated *Bifidobacterium* Bb-12 on the lactose-free yogurt.	Viability of *Bifidobacterium* Bb-12 was found for all spray-dried powders produced with lactose-free skim milk powder, lactose-free skim milk powder and inulin, and lactose-free skim milk powder and oligofructose to be higher than recommended to exert health benefits.	[112]
Lactose-free Greek-style yogurt	Evaluation of potential of lactose-free Greek-style yogurt as probiotic matrix.	Three different microcapsule formulations were produced using gum arabic, inulin and maltodextrin as wall materials. All formulations showed encapsulation yield above 96% and good probiotic viability (>8 log cfu/g) throughout 30 days of storage (4 °C).	[113]
Probiotic Edam cheese	Influence of *Bifidobacterium bifidum* on cheese.	Lactose in control as well as in experimental cheeses (10^7^ viable cell) was depleted within 15 days. The free fatty acids increased from 2.23% and 2.31% on 0-day to 2.78% and 2.83% after 3 months, in control and probiotic cheeses, respectively.	[114]
Lactose-free fermented dairy beverages	Influence of co-cultures of *Streptococcus thermophilus* and probiotic lactobacilli on quality and antioxidant capacity parameters of lactose-free fermented dairy beverages containing *Syzygium cumini* (L.) skeels pulp.	Viability of bacteria are above 7 log CFU/g and total phenolic content around 40 mg GAE/100 g. The dairy beverages are good options for functional foods due to its nutritional value, viability of probiotic lactobacilli, phenolic content, and antioxidant capacity, also serving lactose-intolerant people.	[115]

## Data Availability

No new data were created or analyzed in this study. Data sharing is not applicable to this article.
